# Femtosecond Laser Pulse Ablation of Sub-Cellular Drusen-Like Deposits

**DOI:** 10.1038/s41598-019-52137-1

**Published:** 2019-10-30

**Authors:** Katherine N. Smith, Nir Katchinskiy, Abdulhakem Y. Elezzabi

**Affiliations:** Ultrafast Optics and Nanophotonics Laboratory, Department of Electrical and Computer Engineering, University of Alberta, Edmonton, AB T6G 1H9 Canada

**Keywords:** Ultrafast lasers, Biophotonics

## Abstract

Age-related macular degeneration (AMD) is a condition affecting the retina and is the leading cause of vision loss. Dry AMD is caused by the accumulation of lipid deposits called drusen, which form under the retina. This work demonstrates, for the first time, the removal of drusen-like deposits underneath ARPE-19 cell layers using femtosecond laser pulses. A novel cell culture model was created in response to the limited access to primary cell lines and the absence of animal models that recapitulate all aspects of AMD. In the cell culture model, deposits were identified with fluorescent stains specific to known deposit constituents. Trains of sub-10 femtosecond laser pulses from a Ti:Sapphire laser were used to successfully ablate the deposits without causing damage to surrounding cells. This drusen removal method can be used as a potential treatment for dry-stage AMD.

## Introduction

Many degenerative medical conditions are caused by the accumulation and formation of biological deposits of varied compositions. Age-related macular degeneration (AMD) is one such condition, caused by deposit formation that leads to significant vision loss^1^. AMD is a highly prevalent, degenerative condition of the retina and the current leading cause of vision loss in First World nations^2^. There is no cure; merely methods to slow the progression of the disease.

There are two forms of AMD: dry and wet. Dry AMD is characterized by the development of fatty deposits between the outer layer of the retina, the retinal pigment epithelium (RPE), and Bruch’s membrane, a basement membrane under the RPE^1,3^. These deposits are known as drusen and are comprised of proteins suspended in lipids^4–8^. The severity of dry AMD is determined by the size, shape, and number of drusen present on the retina^9^. Wet AMD is characterized by the growth of new blood vessels underneath the retina. Such vessels originate in the choroid and grow through breaks in Bruch’s membrane and are commonly associated with drusen formation^1,3,8–10^. However, these new blood vessels leak, with exudation of fluid and other blood components underneath the retina, causing further damage. Ninety percent of AMD cases are the dry form, and almost every case of wet AMD develops progressively from advanced cases of dry AMD, rather than developing spontaneously. Thus, if cases of dry AMD can be arrested, development of wet AMD and total vision loss can be prevented. Therefore, saving millions in health care costs and maintaining maximal quality of life for the patient.

Current AMD treatment strategies target the growth of new blood vessels (wet AMD) with anti-vascular endothelial growth factor (anti-VEGF) therapies and nutritional approaches focusing on anti-oxidants, to slow the progression of the condition^1,3^. The current standard is intraocular injections of anti-VEGF to suppress the neovascularization of wet AMD, administered every three to six months, with significant risks and patient discomfort. There is currently no method that can completely restore the retinal function once compromised^8,11^.

A number of laser treatment strategies for including photocoagulation and photodynamic therapy were abandoned due to adverse effects induced by the treatment methods^1,3,12–16^. Photocoagulation for wet AMD used 805 nm diode lasers to cauterize the leaky blood vessels in the retina^14^; however, the laser beam application induced fulminant, untreatable neovascularization recurrences and left large, visible retinal scars^1,12,14^. Additionally, photocoagulation was used for dry AMD as a method to induce drusen resorption with an 810 nm, continuous wave diode laser^15,16^. Similarly, in photodynamic therapy aimed at wet AMD, photosensitive substances are injected systemically, taken up by the vascular tissue, and then activated by the laser beam to induce cell death with thermal energy^13^. This treatment method also leads to wide-scale damage to surrounding healthy tissue as the photosensitive substance is not specific to the targeted tissue and the thermal energy transfers to nearby healthy tissue as well^12–14^. Both previous attempts at laser-assisted treatment were highly non-specific to the destructive drusen or blood vessels. Furthermore, the high levels of collateral healthy tissue damage caused side effects that over-shadowed the treatment potential of these methods. These techniques were abandoned as they did not result in a statistically significant risk reduction. Material ablation with continuous wave (CW) lasers is based on thermal disruption of the material and results in non-localized thermal energy deposition that leads to vast tissue damage. Therefore, a novel laser treatment method using femtosecond (fs) laser pulses aimed at precisely removing drusen without resulting in damage to surrounding tissue is potentially a groundbreaking technique.

Femtosecond laser pulse-tissue interaction has been investigated extensively for a variety of biological applications. Ultrafast laser pulses have been demonstrated as a tool for intra-tissue nano-dissection of single plastids^17^, dissection of fixed and dried metaphase human chromosomes^18^, cell membrane attachment^19^, neuronal attachment^19^, and the ablation of a single mitochondrion^20^. Additionally, fs laser-pulses have been used to non-invasively create reversible pores on the membranes of live cells and zebrafish embryos^21–24^. Femtosecond laser pulses far surpass the tissue interaction capabilities of other laser systems due to the unique non-linear, multiphoton interaction between fs laser-pulses and biological tissue.

For near-infrared femtosecond laser pulse excitation, multiphoton absorption of the photons occurs on the time scale corresponding to the duration of the pulse. This exposure time is much shorter than the time scale of electron-ion energy transfer and the thermal diffusion time. Thus, the electronic excitation is efficiently de-coupled from the thermal relaxation process. Energetic electrons are created locally before they can transfer their energy to the surrounding, minimizing cavitation bubble formation, as well as heat deposition. Above the laser pulse intensity threshold, the electron density grows exponentially since electrons ionized at the leading edge of the femtosecond laser pulse initiate a self-seeding avalanche ionization process. This process creates a high-density plasma that results in a plasma mediated ablation in the targeted material^25,26^. These properties make the fs laser an excellent tool for tissue manipulation as the effects of the laser pulse-tissue interaction are highly confined to the focal spot.

This report describes a method using femtosecond laser pulses to ablate drusen-like sub-RPE deposits in a tissue culture model. The dry AMD cell-culture model was comprised of a human-derived, non-primary cell line that develops sub-RPE deposits. Sub-RPE, drusen-like deposits were removed through laser induced ablation while the overlying cells were unaffected. Successful sub-RPE deposit ablation was achieved with an appropriately selected laser power and exposure time. While previous attempts to treat dry AMD, such as photocoagulation and photodynamic therapy, lead to vast surrounding tissue damage, the fs laser pulse is confined to the focal spot and does not result in damage to surrounding retinal tissue. To our knowledge, this is the first demonstration of fs laser pulse ablation of drusen-like deposits in an *in vitro* model and has major implications for the treatment of dry AMD and other serious degenerative conditions caused by organic deposit accumulation.

**Figure 1:**
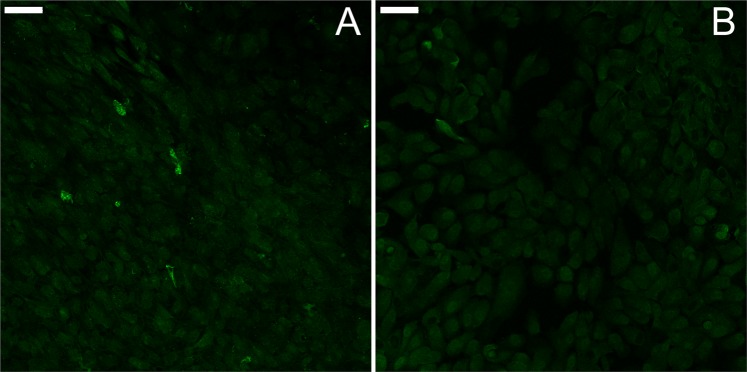
Confocal microscopy images of ARPE-19 cells with ApoE antibody staining grown for (**A**) five weeks, and (**B**) 3 days. Note that the control sample in B shows no deposit immunoreactivity compared to A. The scale bars are 50 μm.

**Figure 2:**
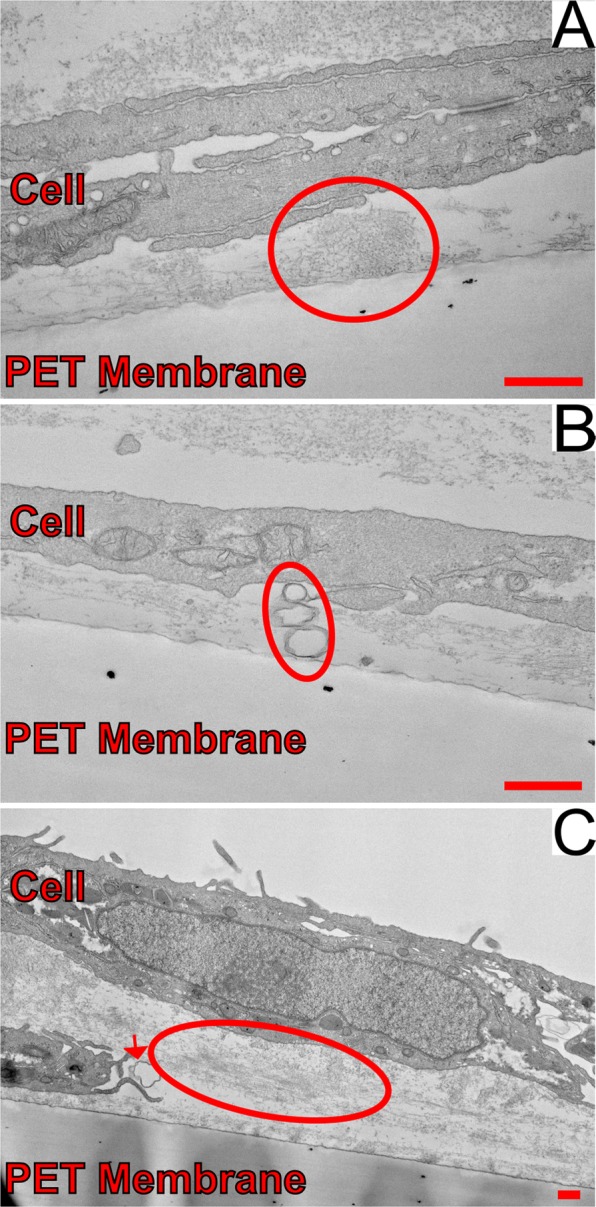
TEM images of sub-RPE deposits formed in a 5-Week ARPE-19 cell culture highlighting (**A**) an area of condensed deposit formation (*circle*), (**B**) examples of membranous deposits (*ellipse*), and (**C**) fibrillar deposit formation (*ellipse*) and a membranous deposit also visible in this area (*arrow*). The scale bars are 500 nm.

**Figure 3:**
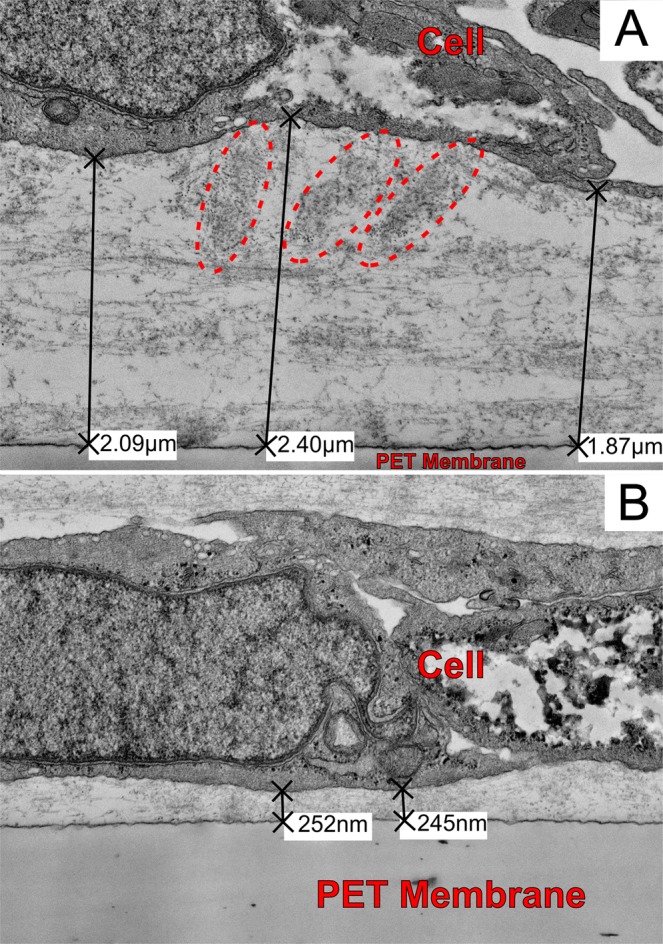
TEM image depicting the distance, as labeled, between the ARPE-19 cell layer and the porous membrane when (**A**) deposits are present (*ellipses*: Condensed deposits) and when (**B**) no deposits are formed.

Few reliable animal models of dry AMD exist, and donor tissue or primary human cells are not readily available to all researchers. Thus, a non-primary culture model using an immortalized cell line of non-primary retinal pigment epithelium cells, ARPE-19^27^ was developed that produces sub-RPE deposits with similar organic composition of naturally occurring AMD drusen. Confocal microscopy of the ARPE-19 cell cultures identified the presence of sub-RPE deposits in samples grown for five weeks. Fluorescent staining showed that ApoE and cholesterol, two major components of drusen, comprise the deposits^4–8,28,29^. Experimental samples that were grown for a minimum of five weeks (5-Week) were compared to cells that were incubated for only a few days (3-Day). Figure 1A shows the experimental sample of 5-Week cells, with several bright spots indicating the sizable sub-RPE deposit accumulation visible through the cell layer. For comparison, a sample of 3-Day cells is shown in Fig. 1B. The 3-Day sample had no regions of fluorescence strength comparable to the deposits in the 5-Week sample. The deposits were stained positively for known drusen components, indicating that drusen-like deposits are present at the 5-Week samples. The sub-RPE deposits in the 5-Week samples were non-uniformly distributed throughout the whole field of view and varied in size, with the largest observed to be approximately 20 µm. Additionally, comparison of a 5-Week sample and a 3-Day sample through transmission electron microscope (TEM) imaging revealed sub-RPE deposits only in the 5-Week sample. Figure 2 shows the different deposit structures observed through TEM. In Fig. 2A, a condensed deposit is highlighted within the circle. Multiple membranous deposits are visible in the circled region of Fig. 2B. The circle in Fig. 2C outlines an area of fibrillar deposit build-up. Additionally, the arrow in Fig. 2C points to a membranous deposit also visible in the same image. Notably, the distance between the RPE cell layer and the porous membrane varied throughout the sample, as shown in Fig. 3. In areas where deposit formation was observed, the cell layer was raised up to 2.4 µm above the membrane (Fig. 3A) due to the accumulated debris. Three regions of fibrillar deposits are outlined by the dotted ovals in Fig. 3A. In contrast, in deposit free areas the cell layer is only 245 nm above the membrane, as seen in Fig. 3B.

**Figure 4:**
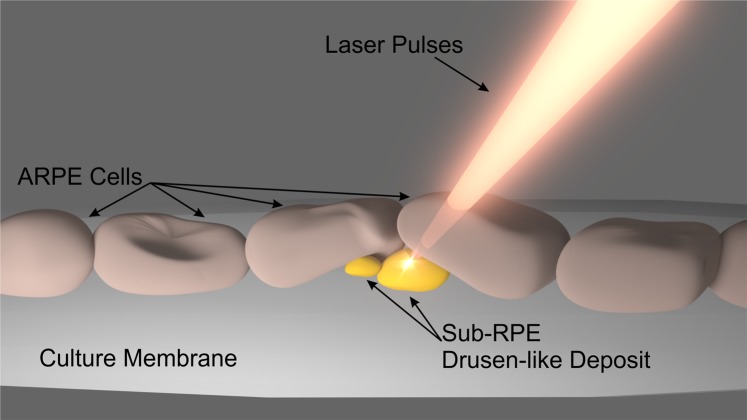
Schematic representation of the laser-tissue interaction between the femtosecond laser pulses and sub-RPE drusen-like deposits. The femtosecond pulse trains pass harmlessly through the ARPE-19. The threshold leading to plasma-induced ablation is only surpassed at the point of focus of the laser, herein located on the drusen-like accumulation.

**Figure 5:**
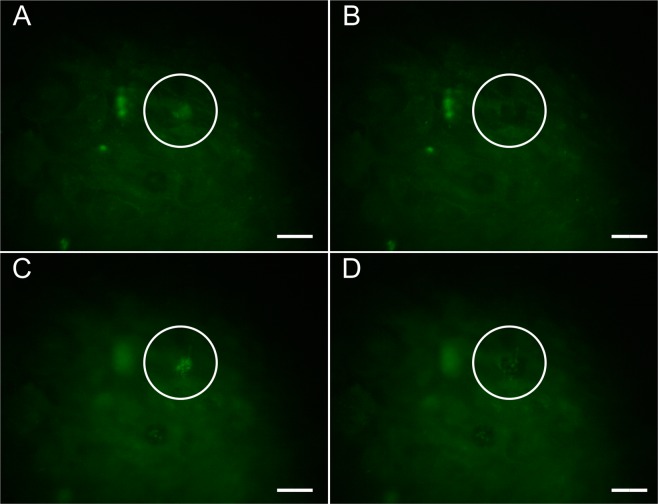
Fluorescence microscopy image, stained for ApoE, of a sub-RPE deposit with the focal plane on the ARPE-19 cell layer (**A**,**B**) and the focal plane on the sub-RPE deposit (**C**,**D**). The fluorescence through the cell layer in (**A**) is that of the deposit in (**C**). After unsuccessful laser ablation a shadow artifact is seen on the cell layer (**B**) and the deposit has been removed (**D**). The scale bars are 10 μm.

Drusen-like sub-RPE deposits in the cell culture model were targeted for ablation with fs laser pulse trains, while the overlying ARPE-19 cells were left untouched. Figure 4 is a schematic representation of the ultrafast laser pulse-tissue interaction during the deposit ablation process. The laser’s intensity and exposure time parameters used in this method are crucial to successfully ablate drusen-like material while leaving the surrounding cells intact. The total exposure time required for complete deposit ablation varied based on the size of the deposit. The laser intensity level was also varied to optimize the ablation of the sub-RPE deposit while preserving the integrity of the overlying RPE cell layer. When excessive energy was used to ablate the drusen-like deposits, visible scars were left on the cell layer above the deposit. Figure 5 depicts the conditions before and after laser application with non-optimized laser parameters. The laser peak intensity was 2.9 × 10^12^ W/cm^2^ and the total exposure time was 1.3 s. The damage to the cell layer from the laser application with non-optimized power and exposure time is evident from the dark shadow (Fig. 5B,D). These artifacts indicate a region of the cell that has been ablated along with the deposit material below the cell layer. As shown in Fig. 5, a combination of high-power level and long exposure time leads to visible damage to the cells overlying the targeted deposit. The damage caused by the laser pulse parameters used in this case is clearly observed when comparing the area before and after laser application.

**Figure 6:**
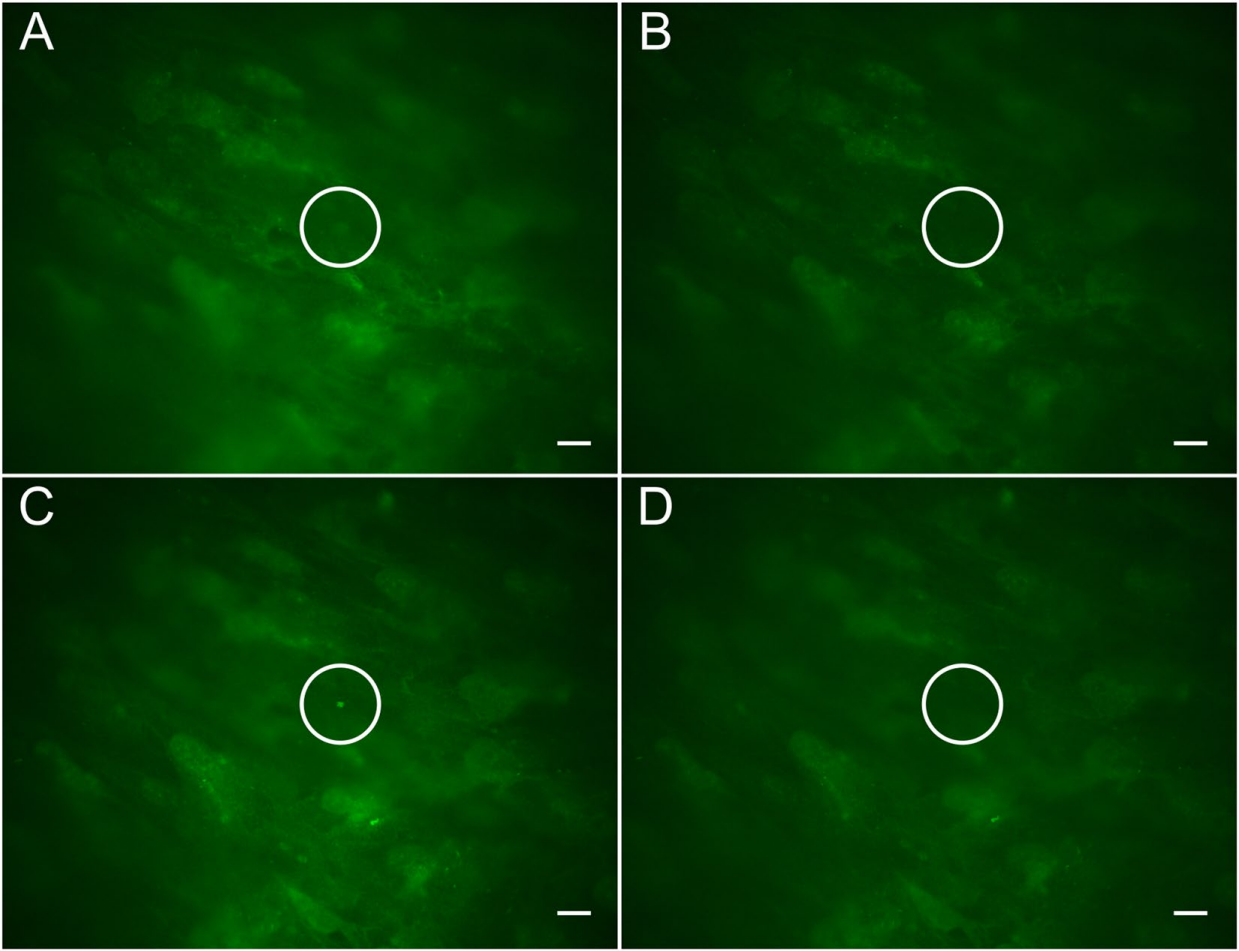
Fluorescence microscopy, using ApoE staining, with the focal plane on the ARPE-19 cell layer (**A** and **B**) and below the cells on a sub-RPE deposit (**C**,**D**). The sub-RPE deposit fluorescence signal is observable through the cell layer (**A**) and in focus (**C**). After laser ablation there is no artifact visible on the cell layer (**B**) and the deposit has been removed (**D**). The scale bars are 20 μm.

Through systematic study changing the power and exposure time, sub-RPE deposit removal without collateral cellular damage was achieved. Figure 6 presents an example of a sub-RPE deposit ablation without collateral damage to the cell layer located above the deposit, at laser pulse train exposure of 150 ms at 2.8 × 10^12^ W/cm^2^ peak intensity. In Fig. 6A, as in Fig. 5A, the focal plane is the cell layer above the sub-RPE deposit before laser treatment. After laser exposure, the drusen-like deposit is no longer visible and the cell layer remains untouched, with no observable shadow or artifact to indicate damage to the cells (Fig. 6B). The sub-RPE deposit targeted for ablation is focused on and observed before ablation in Fig. 6C. After fs laser pulse ablation the deposit is removed (Fig. 6D). The laser tissue interaction was confined within the diffraction limited size of the laser spot, as no tissue damage is visible anywhere outside of the laser focal spot. The successful deposit ablation shown in Fig. 6 is the first example of ultrafast laser pulse ablation of sub-RPE drusen-like deposits in a cell culture model. Due to the low absorption of melanin at 800 nm, the wavelength used in this investigation^30–32^, the laser pulses induce non-linear absorption and material ablation only at the focal spot where its intensity is peaked. Therefore, *in vivo* RPE cells located above drusen would not be affected by the fs laser pulses.

**Figure 7:**
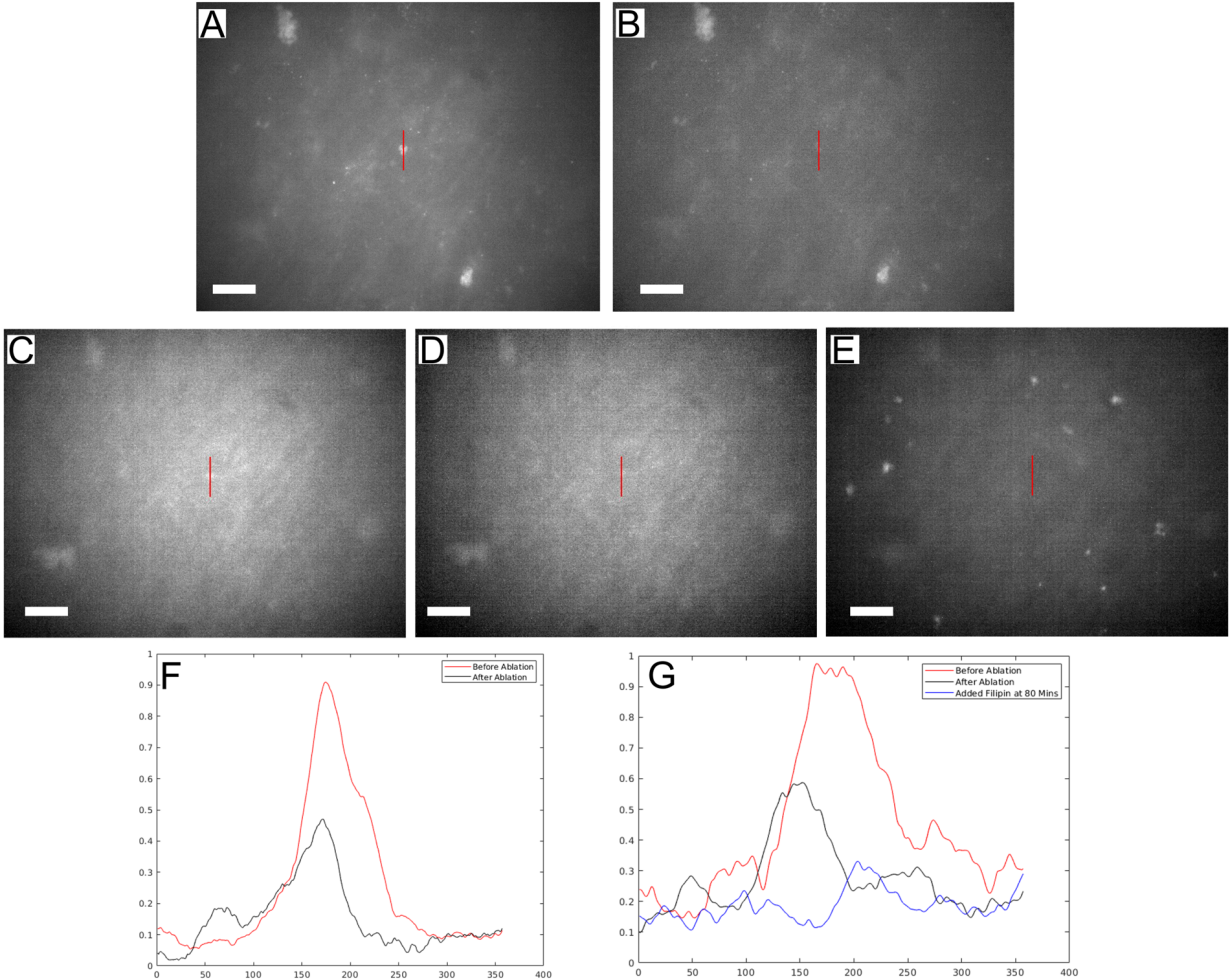
Fluorescence microscopy image of a sub-RPE deposit, stained simultaneously for ApoE (**A**,**B**) and cholesterol (**C**–**E**). (**F**) Line intensity plot shows the profile of the sub-RPE deposit before and after laser ablation, for ApoE. (**G**) Line intensity plot shows the profile of the sub-RPE deposit before and after laser ablation, for cholesterol. (**E**) After laser ablation, the deposit is removed, and additional filipin stain was added after 80 minutes. Areas within the image begin to regain fluorescence, however the deposit area pixel intensity does not return. The red lines in (**A**–**E**) represent the line intensity scan. The scale bars are 20 μm.

To confirm that the deposits were ablated by the fs laser pulses and not removed due to photobleaching of the fluorescent dye, additional filipin stain was added after ablation. Figure 7 shows the fs laser pulse ablation of a sub-RPE deposit that was identified by both its protein and lipid composition with ApoE antibodies and filipin, respectively. ApoE antibodies and filipin were used to identify ApoE lipoproteins and cholesterol, which are two significant components of natural drusen^4–6^. Both stains were used to verify simultaneous lipid and protein composition in the sub-RPE deposits, mimicking the characteristics of *in vivo* drusen. ApoE antibodies provided good contrast while filipin allowed real time addition of more stain to investigate the presence of photobleaching. Figure 7A,B show the deposit using ApoE while Fig. 7C–E are filipin stain images. The deposit prior to fs laser pulse ablation is visible in Fig. 7A,C, while Fig. 7B,D were captured immediately after fs laser pulse ablation. In Fig. 7E, additional filipin stain has been added to the sample and 80 minutes have passed. The deposit in Fig. 7 was ablated at 1.29 × 10^12^ W/cm^2^ peak intensity and a total exposure time of 1.2 s. After the fs laser pulse ablation, more filipin stain was pipetted into the dish to restore fluorescence to photobleached regions. In Fig. 7, a vertical line transect the deposit area, as indicated on each image, and were displayed as a graph of normalized pixel intensity values along that line in Fig. 7F,G. In Fig. 7F, the profile of the deposit is easily identified in the before ablation line-intensity plot. Comparing this line to the after ablation line highlights the removal of the deposit material, as the deposit profile is no longer present. The line intensity plots in Fig. 7G also demonstrates the removal of the drusen from before ablation to after ablation. There is an observable change in the intensity profile in the filipin channel before and after fs laser pulse ablation. Figure 7E represents a threshold of the noise in the image given that this image was taken well after the ablation occurred when there was no remaining deposit material observable in the highlighted area of interest. Thus, the normalized pixel values in the plot here are almost exclusively from background fluorescence noise. It can be clearly shown in Fig. 7E that with additional filipin some accumulations in the frame regained their fluorescent signal. In contrast to these freshly fluorescent spots, the region where the deposit had been before the fs laser pulse application (indicated by the white circle) remained dark indicating the successful ablation of the deposit. The disappearance of the drusen-like deposit was from fs laser pulse ablation and not the photobleaching of the fluorophores used to identify the deposits was verified. The removal of the deposit in Fig. 7 after the fs laser pulse ablation is much more easily observed in the ApoE channel (Fig. 7A,B) over the filipin channel (Fig. 7C,D). However, the filipin images are important in the verification of true ablation over photobleaching. The removal of the deposit from the field of view after the fs laser pulse application was indeed due to the fs laser pulse-induced destruction of the material rather than the photobleaching of the fluorophores within the deposit.

**Figure 8:**
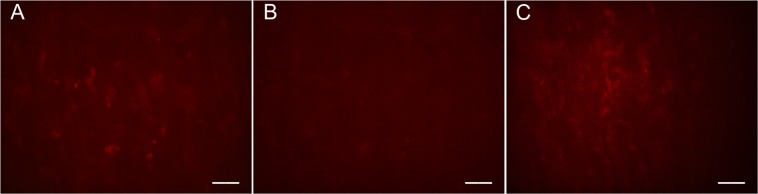
Filipin fluorescence microscopy of an ARPE-19 sample with sub-RPE deposits (**A**) immediately after the excitation shutter was opened and (**B**) the photobleached sample after prolonged and continual excitation source exposure. More filipin stain was added to the dish and after 60 minutes (**C**) some cholesterol rich deposits regain their fluorescence after photobleaching. The scale bars are 20 μm.

The same photobleaching experiment was performed on a sample without fs laser pulse exposure to verify that filipin stained deposits, photobleached from excitation exposure, will regain their fluorescence signal. Figure 8 shows the results of leaving the sample under the excitation source for an extended period until the signal had been significantly reduced, adding more filipin stain to the dish, and waiting 60 minutes after adding the stain. Figure 8A shows the baseline fluorescence of the sample immediately after initial excitation. The sample was left under the open excitation shutter for several minutes until the fluorescence was severely bleached (Fig. 8B). At that point, more filipin was added to the dish and left for 60 minutes (Fig. 8C). This set of images is proof that after photobleaching, adding more filipin will restore visibility of remaining sub-RPE deposits. Thus, when the drusen-like deposit does not return in Fig. 7E, after addition of filipin and time for the stain to react, the conclusion can be drawn that the deposit removal is from the ablative power of the fs laser pulse, not from the effects of photobleaching. The ApoE antibody staining protocol did not permit re-staining while the sample was in the optical setup; thus, photobleaching investigations could only be completed with filipin.

This work demonstrated that sub-RPE drusen-like deposits can be removed using pulses from an ultrafast femtosecond laser. The fs laser pulses can be focused through the RPE cell layer and remove the underlying deposit material while leaving the healthy RPE cells undamaged. The technique demonstrated herein is the first time a fs laser pulses were used for ablation of sub-RPE deposits that mimic those seen in AMD. This fs laser pulse surgery technique for removing drusen deposits while preserving the remaining healthy RPE cells may halt the progression of this degenerative disease. With further research into this new treatment, the millions of patients affected by AMD can potentially be spared from vision loss.

## Materials and Methods

### Cell culture

A custom cell culture model with drusen-like sub-RPE deposit formation using ARPE-19 cells was developed. The non-pigmented ARPE-19 cells were initially seeded 120,000 cells/well onto 0.45 µm pore micro-porous mixed cellulose ester membranes (Millicell-HA PHIA03050; EMD Millipore, Billerica, MA, USA) for confocal microscopy or 0.40 μm pore micro-porous polyethylene terephthalate (PET) membranes (Falcon 353090; Corning Inc., Corning, NY, USA) for TEM imaging. The cells were grown in a media of DMEM F12 supplemented with 10% Fetal Bovine Serum, 2 mM L-glutamine, and 1X Penicillin/Streptomycin. Cells were kept at 37 °C and 5% CO_2_ and the media was changed twice a week during the incubation periods. The experimental cells were grown for a minimum of five weeks before fixation and staining, whereas control cells were grown for less than three days. To prepare the cells, media was decanted, and the cells were rinsed with phosphate-buffered saline (PBS). Fixation was achieved by the addition of 4% paraformaldehyde in PBS, pH 7.4 for 15 minutes at room temperature. Following three washes with PBS the cells were subsequently permeabilized through incubation in 0.2% Triton X-100 in PBS for 10 minutes. After three more PBS washes, cells were left in PBS until staining.

Cells were tagged with fluorescent stains specific to ApoE or cholesterol. For ApoE antibody staining, first, nonspecific binding of the antibodies was blocked by incubating the cells for 30 minutes in 1% Bovine Serum Albumin (BSA) and 22.52 mg/ml glycine in PBS + 0.1% Tween 20 (PBST). Cells were incubated overnight at 4 °C with the primary antibody (Goat polyclonal Anti-Apolipoprotein E, AB947; EMD Millipore, Billerica, MA, USA) diluted to 1/1000 in 3 ml of 1% BSA in PBST. After the overnight incubation, the solution was decanted, and the cells were rinsed with PBS three times for 5 minutes. The secondary antibody (Donkey Anti-Goat IgG H&L – Alexa Fluor 488, AB150129; Abcam, Cambridge, UK) was diluted to 1/500 in 1% BSA in PBST, and the cells incubated for 1 hour in the dark at room temperature. Finally, the cells were washed in PBS three times for 5 minutes while in the dark. To identify cholesterol, cells were stained with Filipin (F9765; Sigma-Aldrich, St. Louis, MO, USA) dissolved in DMSO; the stain was added to the well at a volume of 1/100 for a final concentration of 100 µg/ml. After 1 hour of incubation, the solution was decanted, and the cells were washed with PBS three times for 5 minutes in the dark.

For confocal microscopy, samples were mounted onto glass slides with 1.5 glass coverslips. The porous membranes were excised from the plastic well using a scalpel and the membrane was placed on the slide with the cell side closest to the cover slip. A drop of Prolong Gold Antifade Mounting Reagent (P10144; Invitrogen, Carlsbad, CA, USA), with or without additional DAPI staining, or Permount (SP15-100; Thermo Fisher Scientific, Waltham, MA, USA), depending on the requirements of the imaging session, was placed on the slide above and below the membrane. The slide was left to cure for a minimum of 2 hours and up to overnight. For visualization, excitation of ApoE was at 488 nm and filipin was excited at 405 nm. For laser excitation the membrane was excised from the plastic insert and placed in a glass dish with 5 ml of PBS.

### Electron microscopy

Samples were prepared for investigation by transmission electron microscopy similar to the method outlined by Burles *et al*.^33^ Briefly, cells are grown on PET micro-porous inserts from Falcon before fixation. The fixative was added to the cells and left for an hour at 37 °C. Unlike the Burles *et al*.^33^ protocol, propylene oxide was not used in the preparation of the samples. After cell blocking to improve contrast, cells were embedded in Spurr’s resin and polymerized at 65 °C for 24 hours. Samples were then sectioned with a Leica UC7 ultramicrotome (Leica Microsystems, Inc., Wetzlar, Germany) perpendicularly to the cell layer growth and membrane. Sections were stained with 2% uranyl acetate and Reinolds’ lead citrate then imaged with a Hitachi H-7650 transmission electron microscope (Hitachi High-Technologies, Minato-ku, Tokyo, Japan) and a 16-megapixel TEM camera (XR111; Advanced Microscopy Techniques, MA, USA).

### Fluorescence imaging and laser delivery setup

The fluorescence imaging system and optical setup used is described in detail elsewhere^24,34^. Briefly, the ApoE and filipin stains were observed with standard Endow GFP Longpass (Chroma Technology, En GFP LP 41018) and Quantum Dot 605 (Chroma Technology, Qdot) filters, respectively, mounted in a modified upright Nikon Eclipse 80i microscope. Fluorescence images were captured using the 10 MP Amscope MU1003 camera with the Amscope imaging software. A 60x microscope objective with numerical aperture of 1.0 was used to enable imaging of the cell sample and focusing the laser beam to an approximate spot size of 800 nm. The laser was a titanium sapphire laser oscillator delivering 10 fs pulses at a repetition rate of 80 MHz with a center wavelength of 800 nm; the pulse energy was varied between 1.9–4.2 nJ/pulse. The exposure time was regulated by a computer-controlled, galvanometer-mounted mirror, programed to expose the sample to the laser beam in windows of 15 ms. The total exposure time and pulse energy were varied to determine the optimal ablation parameters, which were evaluated through visual observation of deposit removal.
